# Preventive effects of *Weissella cibaria* CMU on the progression of periodontitis in a rat model

**DOI:** 10.1080/20002297.2025.2469895

**Published:** 2025-02-23

**Authors:** Kyung-Hyo Do, Soyoung Boo, Rayoung You, Sanggu Kim, Soochong Kim, Kwang-Won Seo, Mi-Sun Kang, Wan-Kyu Lee

**Affiliations:** a College of Veterinary Medicine, Chungbuk National University, Cheongju, Republic of Korea; b Laboratory of Veterinary Pathology and Platelet Signaling, College of Veterinary Medicine, Chungbuk National University, Cheongju, Republic of Korea; c R&D Center, OraTicx Inc., Seoul, Republic of Korea

**Keywords:** Periodontal disease(s)/periodontitis, oral hygiene, probiotics, microbiome, inflammation, microbiology

## Abstract

**Background:**

Periodontitis is a widespread chronic inflammatory disease impacting 20–50% of the global population.

**Methods:**

This study evaluated the effects of *Weissella cibaria* CMU (CMU) on preventing the progression of periodontitis in a rat model. Periodontitis was induced by injecting lipopolysaccharide into the palatal gingiva around the first and second maxillary molars. CMU was then administered at three concentrations (low: 2 × 10^7^ CFU/rat/day; mid: 2 × 10^8^ CFU/rat/day; high: 2 × 10^9^ CFU/rat/day) for 14 days to assess its ability to prevent further periodontal damage.

**Results:**

The administration of CMU significantly improved gingivitis and plaque indices in a dose-dependent manner. Macroscopic analysis and micro-computed tomography showed a dose-dependent reduction in alveolar bone loss in the CMU groups. Although histopathological analysis indicated a decrease in bone loss, statistical significance was not achieved in the high-dose group. Pro-inflammatory mediators such as TNF-α, IL-6, MMP-1, and MMP-9 were suppressed in a dose-dependent manner in the CMU groups. Additionally, mid- and high-dose CMU increased the relative abundance of Weissella in the oral microbiome.

**Conclusions:**

CMU can influence the oral microbiome, reduce inflammatory mediators, and alleviate histological changes in periodontal tissue, highlighting its potential as a probiotic strain for preventing periodontitis.

## Introduction

Periodontitis is a prevalent chronic inflammatory condition that affects approximately 20% − 50% of the global population. Notably, periodontal diseases increase the susceptibility to systemic conditions, including cardiovascular diseases [1]. Moreover, triggered by the development of intricate subgingival microbial biofilms, periodontitis is an immune-inflammatory condition that induces inflammatory cells to generate pro-inflammatory cytokines, deteriorating connective tissues, including the subgingiva, periodontal ligament, and alveolar bone [2,3]. Consequently, managing periodontitis through surgical procedures and antibiotic therapy alone may prove insufficient because of the complicated etiological factors associated with the oral microbiota [4].

Notably, several biological substances are recognized for their antibacterial properties, promoting the healing and regeneration of periodontal tissues [5,6]. Particularly, anti-inflammatory medications or inhibitors targeting matrix metalloproteinases (MMPs) and pro-inflammatory cytokines are believed to positively affect periodontal diseases [7]. However, prolonged exposure and/or excessive use of antibiotics may contribute to multiple antibiotic resistance. Moreover, systemic administration of many pharmaceuticals has been shown to induce severe side effects that can lead to complications [8].

Ongoing research is currently exploring the potential use of probiotics to enhance oral health and prevent periodontitis. Probiotics are harmless live microorganisms that confer health benefits and contribute to disease prevention when consumed in sufficient quantities [9]. They exhibit a notable difference in the composition of oral microbiota in individuals with good oral health compared to those with periodontitis. Specific oral lactobacilli, such as *Lactobacillus paracasei* and *Lactobacillus plantarum*, have demonstrated the capability to reduce the occurrence of dental caries by impeding the growth and colonization of cariogenic bacteria [10,11].

Advancements in DNA technology have led to the recent reclassification of *Weissella* into the *Lactobacillus* genus. *Weissella cibaria* is characterized by low acid production and secretion of water-soluble glucan and hydrogen peroxide, which are known to positively contribute to the oral environment by inhibiting the growth of periodontopathogens, like *Fusobacterium nucleatum* and *Porphyromonas gingivalis*, implicated in dental caries and periodontitis [12]. *W. cibaria* has been officially recognized on the food ingredient list of the Korean Food and Drug Administration (KFDA), and the *W. cibaria* CMU (Chonnam Medical University) strain is generally acknowledged as safe (GRAS) by the US FDA. Various oral care probiotics containing *W. cibaria* are commercially available [13–15].

*W. cibaria* CMU isolated from the saliva of healthy children has been suggested as an oral probiotic beneficial to oral health through various studies including *in vitro, in vivo*, and human studies. Several *in vitro* studies have reported that *W. cibaria* CMU has the inhibition of biofilm formation of *Streptococcus mutans* and antibacterial ability against periodontal pathogens [13,16]. In beagle studies, *W. cibaria* CMU was shown to effectively suppress halitosis, reduce plaque index, and inhibit harmful periodontal bacteria [12,17]. In addition, several clinical trials reported that halitosis components were reduced and halitosis of subjects was significantly improved compared to the placebo control group, which was attributed to the oral colonization ability of *W. cibaria* CMU [18,19].

Although many beneficial effects of *W. cibaria* CMU on oral health have been reported to date, the effective mechanism of this strain in preventing periodontitis needs to be clearly elucidated. Therefore, this study aimed to investigate the effect of *W. cibaria* CMU on periodontitis using an experimental mouse model.

## Materials and methods

### Preparation of *W. cibaria* CMU

*W. cibaria* CMU was supplied by OraTicx Inc. (Seoul, Korea). This strain was grown in De Man, Rogosa, and Sharpe (MRS) broth and agar medium (Difco, Detroit, MI, USA), maintained at 37°C for a period of 24 h. After centrifuging the bacterial cells at 8,000 × g for 20 minutes at a temperature of 4°C, the bacterial cell pellet was resuspended in sterile phosphate-buffered saline (PBS) to prepare a bacterial suspension. The concentration of live *W. cibaria* CMU in the suspension was adjusted to the desired levels (1 × 10^8^, 1 × 10^9^, or 1 × 10^10^ CFU/mL) using the plate count method on MRS agar. The bacterial suspension was freshly prepared every three days, stored at 4°C, and its viability was confirmed daily by CFU quantification before administration.

### Experimental animals and environment

The rats underwent a one-week acclimation period before the commencement of the experiments. Male Sprague – Dawley (SD) rats, seven weeks old and weighing 200 ± 20 g, were obtained from KoaTech (Gyeonggi-do, Korea). All animals were certified to be specific pathogen-free and in good health upon arrival. The animals were housed in individually ventilated cages, with each cage housing two rats, under controlled conditions of 21 ± 2°C temperature, 50 ± 5% humidity, and a 12-hour light/dark cycle. Animals were provided with laboratory chow (KoaTech, Gyeonggi-do, Korea) and tap water *ad libitum*. All procedures involving animals were approved by the Institutional Animal Care and Use Committee (IACUC) of Chungbuk National University (approval no. ‘CBNUA-2147-23-02’; approval date: 17 July 2023), in accordance with the ARRIVE guidelines and Korean animal welfare regulations.

### Inducing experimental periodontitis and *W. cibaria* CMU treatment

**Figure 1.**
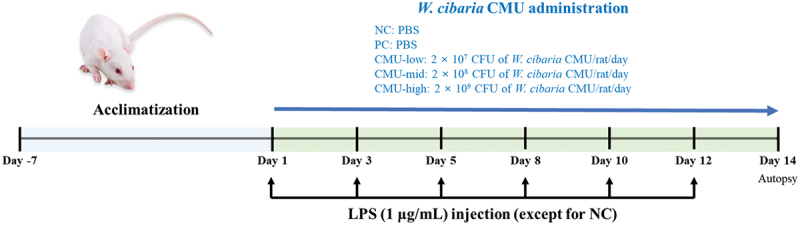
Experimental timeline for periodontitis induction and *Weissella cibaria* CMU administration in a rat model. Periodontitis was induced by injection of LPS (1 μg/mL) into the palatal gingiva around the first and second maxillary molars, three times a week for 14 days. *W. cibaria* CMU was applied to the gingival tissues by syringe daily for 14 days and intragastrically in the same manner. The mice were sacrificed at day 14. NC, negative control; PC, positive control; CMU-low, 2 × 10^7^ CFU; CMU-mid, 2 × 10^8^ CFU; CMU-high, 2 × 10^9^ CFU.

The overall experimental design is illustrated in Figure 1. After a 7-day acclimatization period, the animals were divided into the following groups: negative control (NC), positive control (PC), *W. cibaria* CMU 2 × 10^7^ CFU/rat/day (CMU-low), *W. cibaria* CMU 2 × 10^8^ CFU/rat/day (CMU-mid), and *W. cibaria* CMU 2 × 10^9^ CFU/rat/day (CMU-high), each consisting of eight animals. Over the 14-day experimental period, *W. cibaria* CMU was administered daily using a micropipette. A volume of 100 μL of the suspension was applied directly to the gingival tissues around the first and second maxillary molars, which were the sites of periodontitis induction. Additionally, 100 μL of the suspension was administered intragastrically to evaluate the systemic effects, including pro-inflammatory mediators. The NC and PC groups received an equal volume of PBS applied to the gingival tissues and intragastrically in the same manner.

Periodontitis was induced in the PC group and the CMU-treated groups by injecting 10 μL of lipopolysaccharide (LPS) (*E. coli* O55:B5, 1 μg/μL; Sigma-Aldrich, Darmstadt, Germany) into the palatal gingiva around the first and second maxillary molars under general anesthesia using diethyl ether. Injections were performed using a 30-gauge needle attached to a Hamilton syringe (Hamilton, Reno, NV, USA) and were repeated three times a week for two weeks, targeting both sides of the gingiva to ensure uniform inflammation. The NC group did not receive LPS injections. After inducing experimental periodontitis for 2 weeks, a subset of rats (*n* = 8 per group) was euthanized for subsequent experimental analysis by cervical dislocation following blood collection under diethyl ether anesthesia.

The experimental timeline is as follows: After a one-week acclimation period, periodontitis was induced over two weeks through LPS injections. Simultaneously, *W. cibaria* CMU was administered daily for 14 days starting from the first day of periodontitis induction. At the end of the experimental period, animals were euthanized for sample collection and analysis.

### Measurement of gingivitis and plaque indices

The plaque and gingivitis indices of the rats were measured immediately before euthanasia. Notably, the gingivitis index was assessed using the Löe-Silness gingivitis index, which ranges from 0 (normal gingiva) to 3 (Gingiva is red or reddish-blue, the gingival margin is swollen, tendency to spontaneous hemorrhage or profuse hemorrhage on probing, and ulcerations along the gingival margin) [12]. The buccal gingiva of each evaluated tooth was subdivided into mesial, buccal, and distal regions. Each tooth was graded based on the severity of the three scores obtained per tooth. The overall score for each animal represents the mean score across all evaluated teeth [12].

The plaque index was determined using the Logan – Boyce modification plaque index [12]. After applying a dental disclosing solution (Erythrosin; Sultan Chemists, Englewood, CA, USA) to the teeth, the dental surfaces were horizontally divided into gingival and occlusal sections. Each section was scored based on coverage (0–4) and thickness (1–3) of the plaque. The coverage score represents the proportion of the tooth surface covered by plaque (0: no plaque, 4: >75% coverage), while the thickness score indicates the density of the plaque (1: light, 3: heavy). The final plaque index for each tooth was calculated by multiplying the coverage and thickness scores, and the mean score across all teeth was used to represent the whole-mouth plaque index [12].

### Measurement of alveolar bone loss: macroscopic analysis

After euthanasia, the maxilla of each animal was dissected and made hemispherical. The left maxillary sample was used for the macroscopic analysis of alveolar bone loss. The gingival tissues surrounding the maxillary molars were carefully removed, and the bone samples underwent a 24-hour immersion in 3% hydrogen peroxide to facilitate the removal of any remaining soft tissue. Subsequently, the samples were stored in 70% ethanol until the macroscopic evaluation of alveolar bone loss. The bone resorption areas on the buccal and palatal surfaces of the first maxillary molars were measured macroscopically. Briefly, bone samples were extracted with 70% ethanol, dried, and immersed for 5 min in a methylene blue solution (0.7 g/L). Afterward, the samples were rinsed with water to remove the excess methylene blue. Digital photographs of the buccal and palatal surfaces of the stained samples were captured using a stereomicroscope at 20× magnification (Bhattari et al., 2016). The measurement of the distance between the cemento-enamel junction (CEJ) and the alveolar bone crest (ABC) of the first molar was conducted at three distinct points (corresponding to the three roots of the first molar) utilizing ImageJ software (NIH). Alveolar bone loss was determined by calculating the average distance from the CEJ to the ABC across the three roots.

### Measurement of alveolar bone loss: microcomputed tomography scanning

Following macroscopic analysis, the dissected maxillary samples were meticulously fixed in a 10% neutral formalin solution for 2 d. Subsequently, they were stored in 70% ethanol before undergoing computed tomography scanning. The maxillae were scanned using SKYSCAN 1273 (Bruker-microCT, Kontich, Belgium) under the following operational conditions: Al 1.0 mm of filter, 75 kV of source voltage, 53 μA of source current, and 1.0 degree of rotation step. The scanned data of the maxillary samples were analyzed using DataViewer (version 1.7.0.1, Bruker) and CTVox (version 3.3.0, Bruker). Alveolar bone loss was determined using the same criteria as those employed in the macroscopic analysis.

### Histopathologic evaluation

The right maxillary bone was immersed in formalin for 2 days, decalcified with a Shandon TBD-1 decalcifier for 12 h, and then embedded in paraffin following established protocols. Formalin-fixed paraffin-embedded blocks were sectioned into 5 μm-thick slices using a tissue slicer. The tissue sections were stained with Hematoxylin and Eosin. All slides were captured and analyzed using an Olympus VS200 Virtual Slide System (VS200, Olympus, Tokyo, Japan). The extent of alveolar bone loss and periodontal ligament degeneration in the periodontal tissue of the right upper maxilla was assessed on a scale from 0 to 3 based on severity: 0 = minimal; 1 = mild; 2 = moderate; and 3 = severe. The criteria for evaluating the degree of alveolar bone loss were as follows: 1) alveolar bone loss (osteoclastic resorption) with increasing osteoclast activity, 2) vertical bone loss and triangular-shaped defects in the apex of the alveolar bone, 3) an increase in the distance from the CEJ to the apex of the alveolar bone (AB), and 4) fibrous connective tissue proliferation in the area of alveolar bone loss. The criteria for evaluating the degree of periodontal ligament degeneration included: 1) inflammatory cell infiltration in the periodontal tissue, 2) an increased number of newly formed blood vessels, and 3) large areas of collagen-depleted connective tissue in the periodontium.

### Enzyme-Linked Immunosorbent Assay (ELISA)

For the quantification of interleukin (IL)-1β, tumor necrosis factor (TNF)-α, MMP-1, and MMP-9 levels, a rat ELISA kit from Invitrogen (Waltham, MA, USA) was employed, following the assay protocol provided by the manufacturer. Each sample and standard were tested in triplicates. The absorbance was measured at 450 nm using a Tecan Sunrise ELISA reader (Männedorf, Switzerland). The concentration of each variable was determined based on the corresponding standard curve.

### Measurement of oral bacterial community and bioinformatic analysis

To measure the oral bacterial communities in each group, four rats per group were randomly selected. Right before the euthanasia, the oral swabbed samples from each animal were gathered in sterile containers and subsequently preserved at −70°C. DNA from bacteria was extracted from samples collected through oral swabs with the assistance of a Maxwell RSC Instrument (Promega, Madison, WI, USA) alongside a Maxwell RSC Fecal Microbiome DNA Kit AS1700 (Promega), in accordance with the instructions provided by the manufacturer. Following extraction, the quality of the DNA was evaluated employing a Qubit dsDNA HS assay kit (Life Technologies, Gent, Belgium) through a Qubit fluorometer (Thermo Fisher Scientific), adhering to the guidelines provided by the manufacturer.

Following the DNA quantification, twenty libraries consisting of 16S V3-V4 amplicons (with four samples in each group) were generated in compliance with the Illumina metagenomic sequencing library preparation protocol (Part #15,044,223 Rev. B, Illumina, San Diego, CA, USA). To summarize, the V3–V4 region of the 16S rRNA bacterial gene was amplified utilizing a KAPA HiFi HotStart ReadyMix PCR Kit (KAPA Biosystems, Wilmington, MA, USA) along with specific primers (16S rRNA gene-specific sequences were underlined): forward 5'-TCGTCGGCAGCGTCAGATGTGTATAAGAGACAGCCTACGGGNGGCWGCAG-5' and reverse 5'-GTCTCGTGGGCTCGGAGATGTGTATAAGAGACAGGACTACHVGGGTATCTAATCC-3'. Subsequently, a second PCR was performed to attach index adapters for the purpose of sample multiplexing, employing a Nextera XT Index Kit (Illumina). The DNA library concentration was measured with a Qubit fluorometer (Thermo Fisher Scientific). DNA libraries were pooled to achieve a concentration of 4 nM and subsequently denatured using 0.2 N NaOH. The ultimate concentration of the library reached 7 pM, with the phiX control library constituting 30% v/v of the samples prepared for loading, matched at the same concentration of 7 pM as the main library. Library sequencing was performed on a MiSeq platform following the manufacturer’s instructions using a MiSeq Reagent Kit v3 (600 cycles) (Illumina).

The 16S rRNA gene sequences were acquired and analyzed through the classify-sklearn naïve Bayes classifier workflow within QIIME 2 version 2023.7. The reads were demultiplexed and trimmed, and forward and reverse reads were merged and matched to their respective samples based on their assigned indices. Following this, raw reads were trimmed utilizing QIIME, and quality control was enforced on the merged sequences. This involved discarding sequences failing to adhere to the specified length requirement of being less than 20 or more than 300 nucleotides. Chimeric sequences were identified and eliminated, with the remaining sequences being clustered into operational taxonomic units (OTUs) using the SILVA v138 database at 99% similarity for full-length sequences. Before the calculation of alpha and beta diversity metrics, the sequence data underwent rarefaction. The adequacy of the sample size and the estimation of species richness were assessed through the construction of a rarefaction curve. The assessment of species richness and evenness, with consideration for sampling depth, was conducted using the Chao 1 alpha diversity index in QIIME 2.

For beta diversity analysis, Bray – Curtis distances were calculated, followed by principal coordinate analysis. Differences in microbial diversity across samples (beta diversity) were measured by Bray – Curtis distances and depicted through multidimensional scaling plots for visual representation. A multifactorial permutational analysis of variance (PERMANOVA) was utilized to investigate the relationship between each experimental group, employing QIIME 2 version 2023.7 (http://www.qiime2.org/, accessed on 22 November 2023).

The abundance of intestinal bacteria was examined via MicrobiomeAnalyst (available at https://www.microbiomeanalyst.ca/), which was accessed on 22 November 2023. Cluster analysis was used to differentiate between high- and low-abundance taxa, with color gradients indicating the degree of similarity in community composition among the various samples. Cluster analysis based on the top 37 genera was conducted using Ward’s method and a Euclidean distance matrix. Clustering outcomes were integrated with the relative abundance data of the species at the genus level within each sample.

### Statistical analysis

Statistical differences among the experimental groups were assessed using analysis of variance (ANOVA) and subsequently compared using Duncan’s post hoc tests using SPSS Statistics version 21.0 software (IBM Corp., Armonk, NY, USA). Statistical significance was set at *p* < 0.05.

## Results

### Effect of *W. cibaria* CMU on gingivitis and plaque indices

**Figure 2.**
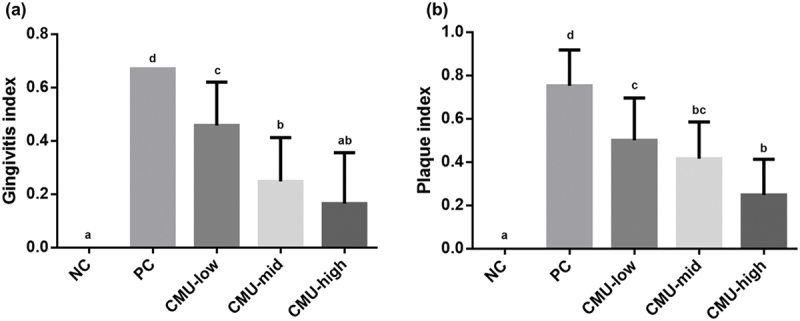
Gingivitis index (a) and plaque index (b) of each experimental group after 14 days inducing experimental periodontitis. NC, negative control; PC, positive control; CMU-low, 2 × 10^7^ CFU; CMU-mid, 2 × 10^8^ CFU; CMU-high, 2 × 10^9^ CFU. Data were expressed as mean ± standard deviation. Different alphabet letters (a, b, c and d) indicate the statistical differences determined by ANOVA (*p* < 0.05).

Following the evaluation of the gingivitis index two weeks after inducing periodontitis (Figure 2a), the NC group recorded 0.00 ± 0.00, indicating the absence of gingivitis in all subjects, while the PC group showed a significant gingivitis occurrence value of 0.67 ± 0.00 (*p* < 0.05). When *W. cibaria* CMU was administered at various concentrations for 14 d, the gingivitis index significantly decreased in all groups compared to the PC group (*p* < 0.05): low dose group (CMU-low; 0.46 ± 0.16), medium dose group (CMU-mid; 0.25 ± 0.17), and high dose group (CMU-high; 0.17 ± 0.19).

During the evaluation of the plaque index 2 weeks post periodontitis induction (Figure 2b), the NC group recorded 0.00 ± 0.00, indicating the absence of plaque formation in all subjects, whereas the PC group measured 0.75 ± 0.17, revealing a significant occurrence of plaque (*p* < 0.05). Upon administering *W. cibaria* CMU at various concentrations for 2 weeks, the plaque index decreased significantly in all cases compared to that in the PC group (*p* < 0.05): CMU-low: 0.50 ± 0.20, CMU-mid: 0.42 ± 0.17, and CMU-high: 0.25 ± 0.17.

### Effect of *W. cibaria* CMU on alveolar bone loss

**Figure 3.**
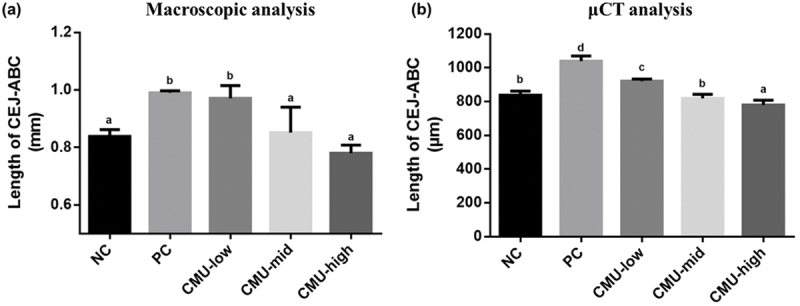
Distance from cemento-enamel junction (CEJ) to alveolar bone crest (ABC) of each experimental group via macroscopic analysis (a) and micro-CT analysis (b) after 14 days inducing experimental periodontitis. NC, negative control; PC, positive control; CMU-low, 2 × 10^7^ CFU; CMU-mid, 2 × 10^8^ CFU; CMU-high, 2 × 10^9^ CFU. Data were expressed as mean ± standard deviation. Different alphabet letters (a, b, c and d) indicate the statistical differences determined by ANOVA (*p* < 0.05).

In the assessment of the CEJ-ABC distance in SD rats through macroscopic analysis, 2 weeks after inducing periodontitis (Figure 3a), the CEJ-ABC distance of the NC group, receiving no treatment, measured 0.84 ± 0.02 mm. On the other hand, the PC group recorded 0.99 ± 0.01 mm, indicating a significant loss of alveolar bone in the PC group (*p* < 0.05). Upon administering *W. cibaria* CMU at different concentrations for 2 weeks, the CEJ-ABC distance of the CMU-low measured 0.97 ± 0.04 mm, showing no significant difference compared to the PC group. However, the CEJ-ABC distances of the CMU-mid and the CMU-high groups were 0.85 ± 0.09 mm and 0.78 ± 0.03 mm, respectively (*p* < 0.05). These values were not significantly different from those of the NC group and showed a significant difference compared to those of the PC group.

Upon evaluating the CEJ-ABC distance of SD rats through Micro-CT analysis 2 weeks after periodontitis induction (Figure 3b), the CEJ-ABC distance of the NC group, receiving no treatment, measured 838.75 ± 23.40 μm. In contrast, the PC group recorded 1,039.17 ± 30.70 μm, indicating a significant loss of alveolar bone in the PC group (*p* < 0.05). Furthermore, administration of *W. cibaria* CMU at different concentrations for 2 weeks resulted in significantly lower CEJ-ABC distances in all administered groups compared to those in the PC group (*p* < 0.05): CMU-low: 921.67 ± 12.29 μm, CMU-mid: 819.67 ± 24.28 μm, and CMU-high: 779.33 ± 28.72 μm.

### Evaluation of periodontal histopathological impact of *W. cibaria* CMU

**Figure 4.**
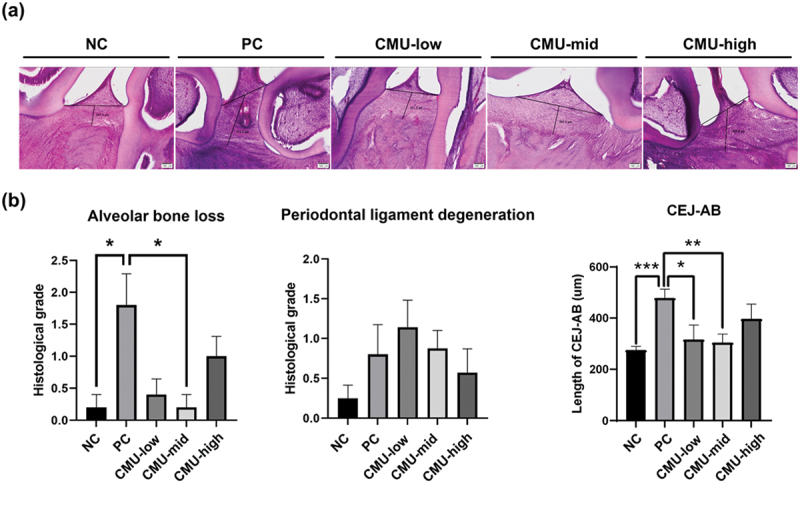
Representative histological findings of periodontal changes. (a) Histological images revealed the effect of *W. cibaria* CMU on alveolar bone loss and periodontal ligament degeneration induced by LPS for 14 days. (b) Histopathologic examination of periodontal changes was scored using hematoxylin and eosin staining. NC, negative control; PC, positive control; CMU-low, 2 × 10^7^ CFU; CMU-mid, 2 × 10^8^ CFU; CMU-high, 2 × 10^9^ CFU. Data are expressed as mean ± standard error. *p < 0.05, **p < 0.01, ***p < 0.001.

In the histopathological analysis of the group subjected to 2 weeks of experimental periodontitis induction (Figure 4), the periodontal tissues in the NC group exhibited a normal histological structure, with a measured CEJ-AB length of 274.8 μm. Conversely, in the PC group, a notable alveolar bone loss was observed, accompanied by mild periodontal ligament degeneration, leading to a 74% increase in the CEJ-AB length to 478.6 μm (*p* < 0.05). In comparison to the PC group, the CMU-low and -mid groups showed a significant reduction in alveolar bone loss, with corresponding CEJ-AB lengths of 316.0 μm and 303.9 μm, respectively (*p* < 0.05). However, the CMU-high group exhibited only mild alveolar bone loss, and the CEJ-AB length was slightly reduced to 396.3 μm. There was no significant difference in periodontal ligament degeneration among the NC, PC, and CMU groups.

### Effect of *W. cibaria* CMU on pro-inflammatory mediators levels

**Figure 5.**
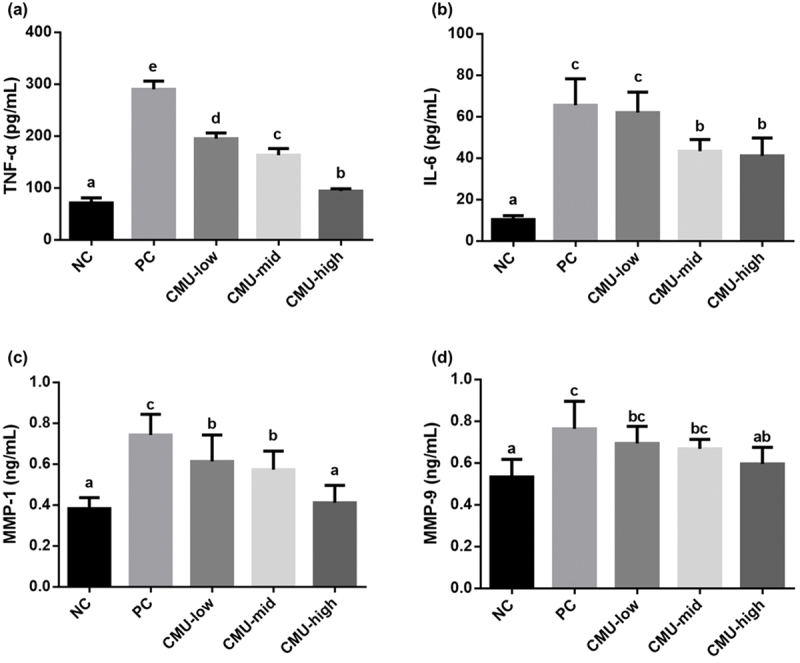
The level of TNF-α (a), IL-6 (b), MMP-1 (c), and MMP-9 (d) in SD rat model in each experimental group after 14 days inducing experimental periodontitis. NC, negative control; PC, positive control; CMU-low, 2 × 10^7^ CFU; CMU-mid, 2 × 10^8^ CFU; CMU-high, 2 × 10^9^ CFU. Data were expressed as mean ± standard deviation. Different alphabet letters (a, b, c, and d) indicate the statistical differences determined by ANOVA (*p* < 0.05).

The results of evaluating the levels of pro-inflammatory mediators at 14 d after periodontitis induction are shown in Figure 5. The CMU-low (194.87 ± 10.82 ρg/mL), CMU-mid (163.33 ± 12.45 ρg/mL), and CMU-high (93.70 ± 4.83 ρg/mL) groups had lower concentrations compared to the PC group (290.10 ± 15.78 ρg/mL). A significant decrease was observed in TNF-α dose-dependently (*p* < 0.05) (Figure 5a). Regarding IL-6, there was a statistically significant decrease in the CMU-mid and high groups (41.08 ± 8.58 ρg/mL) compared to the PC control group (65.50 ± 12.81 ρg/mL) (*p* < 0.05) (Figure 5b). Upon assessing MMP-1 levels, a notable reduction was also detected in the groups treated with *W. cibaria* CMU, which occurred in a concentration-dependent fashion (CMU-low, 0.61 ± 0.13 ng/mL; CMU-mid, 0.57 ± 0.57 ng/mL; CMU-high, 0.41 ± 0.09 ng/mL) (*p* < 0.05) (Figure 5c). Likewise, on measuring MMP-9 levels, a statistically significant decrease was observed in the CMU-high group (0.64 ± 0.09 ng/mL) compared to the PC control group (0.80 ± 0.14 ρg/mL) (*p* < 0.05) (Figure 5d).

### Effect of *W. cibaria* CMU on the oral microbiota

The outcome of computing the relative abundance of the predominant oral microflora at the phylum (Supplementary Figure S1a), class (Supplementary Figure S1b), and order (Supplementary Figure S1c) levels 14 days after periodontitis induction. A comparative analysis of the distribution rates of the predominant bacterial species at each level revealed no significant differences.

**Figure 6.**
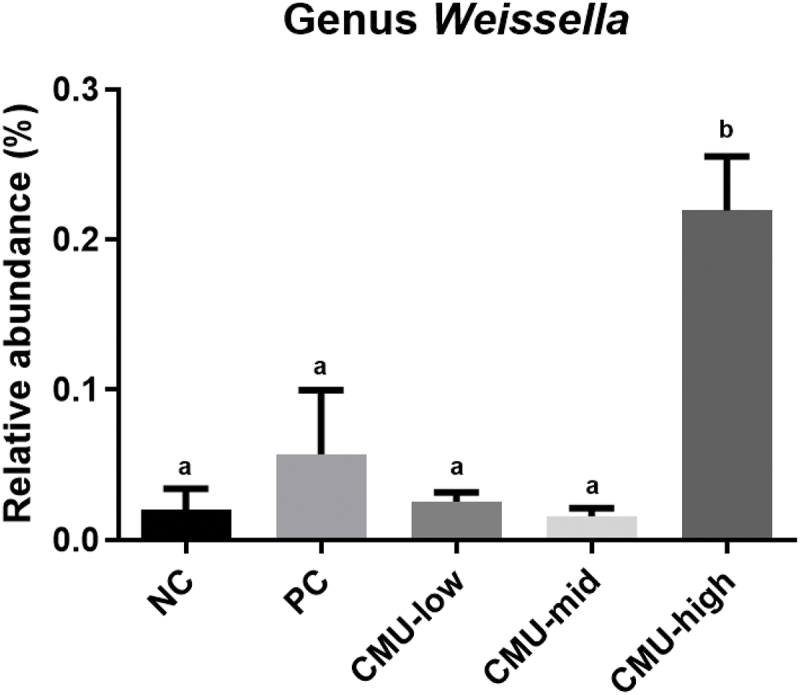
The relative abundance (%) of *Weissella* of oral swab samples in SD rat model in each experimental group after 14 days inducing experimental periodontitis. NC, negative control; PC, positive control; CMU-low, 2 × 10^7^ CFU; CMU-mid, 2 × 10^8^ CFU; CMU-high, 2 × 10^9^ CFU. Data were expressed as mean ± standard deviation. Different alphabet letters (a, and b) indicate the statistical differences determined by ANOVA (*p* < 0.05).

Figure 6 displays the findings from the analysis of the relative abundance of only the genus *Weissella*, the genus of the test strain. The analysis revealed that the distribution rate of the *Weissella* genus in the CMU-mid group was 0.16 ± 0.01%, whereas, in the CMU-high group, it reached 0.22 ± 0.04%, indicating an increment of 0.14% and 0.20%, respectively, compared to the NC group (0.02 ± 0.01%). Statistical analysis revealed no significant differences among the NC, PC, CMU-low, and CMU-mid groups. However, the CMU-high group exhibited a significantly higher relative abundance compared to all other groups (*p* < 0.05).

After 14 days of periodontitis induction, the alpha diversity of the oral microflora in each experimental group was assessed at the feature level using the Shannon index. Statistical analysis using the Mann – Whitney/Kruskal – Wallis method (Supplementary Figure S2a) revealed no significant differences in alpha diversity among the experimental groups. Furthermore, a species diversity analysis (beta diversity) of the oral microflora was performed for each experimental group using the Bray – Curtis index at the feature level (Supplementary Figure S2b), and no significant differences were observed among the experimental groups.

## Discussion

Periodontitis is a chronic inflammatory disease prevalent worldwide [1]. Research has indicated that the systemic administration of various pharmaceuticals is known to induce severe side effects and complications [8]. To address this challenge, probiotics have emerged as an innovative treatment for periodontitis and offer alternative therapeutic approaches.

Lactic acid bacteria (LAB) are known for their diverse beneficial functions, including their ability to alleviate gut-related issues and modulate systemic immunity [9]. While LAB have traditionally been limited to digestive system applications, they have recently gained attention as probiotics, providing an appealing option to replace conventional drugs for enhancing oral health. Studies have demonstrated that *Bifidobacterium* and *Lactobacillus reuteri* can reduce *S. mutans* levels, a recognized contributor to dental caries [20,21]. Additionally, Hatakka et al. [22] demonstrated that *Lactobacillus rhamnosus* GG suppresses *Candida albicans*. Likewise, the *W. cibaria* CMU strain used in this study has been shown to inhibit the growth of bacteria associated with periodontal disease *in vitro* [13,16]. Previous studies have shown the strain’s susceptibility to common antibiotics, with potential exceptions for kanamycin and vancomycin, and its inability to transfer antibiotic resistance to other bacteria [14]. Further investigations by Kang et al. [14] revealed that *W. cibaria* CMU is a safe strain that lacks virulence genes associated with pathogenic bacteria and does not induce hemolysis, mucin or proteolysis, platelet aggregation, or the production of D-lactate or urease.

Plaque, a microbial film on tooth surfaces, plays a pivotal role in the onset of dental caries and periodontal disease. In a beagle model, Do et al. [12] reported that plaque formation can lead to a roughened tooth surface within a few days, facilitating the binding of organic compounds to salivary minerals and contributing to calculus formation. Given that calculi are a major factor in the induction of periodontitis, preventive measures against plaque formation are crucial in averting periodontal issues.

In this study, we administered the probiotic *W. cibaria* CMU to an experimental rat model of periodontitis and evaluated its effect on the plaque index. We found that the groups treated with *W. cibaria* CMU exhibited significantly lower plaque indices than the PC group. Consistent with this, Kang et al. [23] reported through an *in vitro* study that *W. cibaria* hinders plaque formation by producing water-soluble glucans. Additionally, it has been reported that *W. cibaria* CMU reduces the plaque index when administered to beagles for 6 weeks [12,17]. Thus, this study further supports previous results by demonstrating that *W. cibaria* CMU significantly reduces the plaque index in an experimental periodontitis-induced rat model.

Our results showed that the administration of *W. cibaria* CMU lowered the gingivitis index compared to the PC group. Gingivitis is a critical precursor of periodontitis, and when left untreated, inflammation progresses beyond the gingiva to involve the deeper structures supporting the teeth [24]. The primary cause of gingivitis is the presence of plaque, a bacterial film on the teeth and gingivae. Mild inflammation can lead to the formation of pockets between teeth and gingivae, providing an environment for harmful bacteria to thrive. These bacteria trigger an immune response, causing further inflammation and damage to surrounding tissues [25]. Thus, addressing and managing gingivitis through proper oral hygiene practices and professional dental care are crucial for preventing the onset and progression of severe periodontal disease. Therefore, the alleviation of gingivitis by *W. cibaria* CMU is a significant result because it contributes to preventing the onset and progression of severe periodontal disease.

Interestingly, our findings revealed the protective effect of *W. cibaria* CMU on the periodontium. Macroscopic, micro-CT, and histological analyses collectively demonstrated that the administration of *W. cibaria* CMU mitigated alveolar bone loss in an experimental rat model of periodontitis. While our results demonstrated significant reductions in gingivitis and plaque indices, as well as alveolar bone loss, these findings reflect the mitigation of disease progression rather than outright prevention. The protective effects observed are likely mediated through the anti-inflammatory activity and modulation of the oral microbiota by *W. cibaria* CMU. The differences observed between the CEJ-ABC measurements (Figure 3) and the histological evaluations (Figure 4), particularly in the CMU-mid and CMU-high groups, can be attributed to methodological variations between the two analyses. Despite these methodological differences, both approaches consistently demonstrated the protective effects of *W. cibaria* CMU in mitigating alveolar bone loss and reducing tissue damage caused by periodontitis. This protective effect is believed to be attributed to the anti-inflammatory activity of *W. cibaria* CMU [26]. A recent study validating that *W. cibaria* administration alleviates alveolar bone loss and mitigates periodontal tissue damage in an experimental mouse model of periodontitis strongly supports our findings [27].

Pro-inflammatory cytokines, such as TNF-α and IL-6, elicited by LPS, are recognized contributors to periodontal tissue destruction [27]. Twetman et al. [28] demonstrated that the probiotic *L. reuteri* can reduce TNF-α and IL-6 levels, aligning with our current data and findings from previous studies [28,29]. In the current study, as periodontitis was induced, a significant increase in the production of pro-inflammatory cytokines TNF-α and IL-6 was observed. Importantly, the administration of *W. cibaria* CMU markedly reduced TNF-α and IL-6, indicating its inhibitory effects on pro-inflammatory cytokine production.

Periodontal tissues respond to inflammatory mediators by increasing MMP production [30,31]. The secretion of inflammatory mediators, such as pro-inflammatory cytokines and MMPs, triggers alterations in connective tissue and bone metabolism. This process results in the breakdown of the periodontal ligament and the resorption of alveolar bone [16]. Consequently, inhibiting the expression of these inflammatory mediators is a promising strategy for preventing periodontal diseases. MMPs, specifically collagenase (MMP-1) and gelatinase (MMP-9), have been implicated in the progression of periodontitis. In this study, administration of *W. cibaria* CMU resulted in a dose-dependent reduction in MMP-1 and MMP-9 levels. Thus, in conjunction with previous research, our findings suggest that the administration of *W. cibaria* CMU has multiple beneficial effects in suppressing alveolar bone loss, attributed to its anti-inflammatory activities.

In this study, we investigated the changes in the oral microbial composition when *W. cibaria* CMU was administered concurrently with the induction of periodontitis in rats. The significance of the microbiome in relation to disease has gained increasing attention in recent years [32]. Alpha diversity characterizes the distribution of species abundance within a specific sample, considering both species richness and evenness. Beta diversity was used to examine differences in species composition among various samples and habitats. In this study, the administration of *W. cibaria* CMU did not affect alpha and beta diversity. This is presumed to be due to the extended period required, approximately 2–3 months or more, for significant changes to manifest in the oral microbiome [33]. The oral microbiome is a sophisticated and intricate ecosystem characterized by dynamic and multifaceted relationships. Within this intricate microbial community, a diverse array of microorganisms coexist and engage in nuanced interactions with each other and the host. These relationships within the microbiome transcend simple interactions and encompass elaborate networks of symbiosis, competition, and communication.

While no statistical significance was observed in the alpha and beta diversities, it is noteworthy that the groups administered *W. cibaria* CMU exhibited considerably higher values of *Weissella* than the NC and PC groups, aligning with the findings from previous studies. *W. cibaria* CMU strongly adheres to epithelial cells *in vivo* and *in vitro* through the proteinaceous components on its surface [12,34]. Moreover, *W. cibaria* CMU has been demonstrated to impede the growth of oral pathogenic bacteria through mechanisms such as competitive adhesion inhibition, coaggregation, bacteriocin production, and immune regulation [12,16,34]. Thus, *W. cibaria* CMU holds promise for improving oral health.

The limitations of this study are primarily related to the methodology used. Firstly, we did not perform microbial confirmation, and the absence of microbial confirmation through culture and identification hinders a comprehensive understanding of the oral microbiota in individuals with periodontitis. Secondly, the characterization of microbial communities relied only on the MiSeq Illumina platform, focusing on the common V3–V4 fragments of the 16S rRNA gene. It is essential to note that the choice of the region (e.g. V1–V9) within the 16S rRNA gene may introduce variations in different microbial taxa [35]. Although an increased relative abundance of the genus *Weissella* was observed in CMU-treated groups, this genus includes multiple species. The low abundance and lack of species-specific identification in this study prevent definitive conclusions regarding the colonization of *W. cibaria* CMU. Future studies employing strain-specific analyses, such as qPCR, are warranted to confirm its colonization potential. Finally, employing primers that specifically target certain bacterial groups can restrict the identification and targeting of additional microbial groups, possibly affecting the ability to compare results between different studies. Consequently, future research could benefit from exploring bacterial species, their functions, and their correlations with host genetic factors.

In conclusion, the efficacy of the probiotic *W. cibaria* CMU in mitigating the progression of periodontitis was assessed in an SD rat model of LPS-induced periodontitis. Evaluation of clinical index-related parameters revealed that gingivitis and plaque indices significantly reduced in a dose-dependent manner in the groups administered *W. cibaria* CMU at all concentrations. Specifically, the administration of medium and high doses of *W. cibaria* CMU significantly prevented alveolar bone loss, as indicated by the assessment of periodontitis-related indicators. Furthermore, ELISA analysis of periodontitis-related markers in the blood showed that *W. cibaria* CMU exhibited significant preventive ability to induce periodontitis in TNF-α, IL-6, MMP-1, and MMP-9 categories. Histopathological evaluation revealed an improvement in periodontitis in groups administered *W. cibaria* CMU. Additionally, we observed an increased relative abundance of the genus *Weissella* in the oral microbiome of CMU-high group using next-generation sequencing, suggesting a potential colonization effect of *W. cibaria* CMU, though definitive evidence of strain-specific colonization remains to be demonstrated. Therefore, *W. cibaria* CMU mitigates the progression of periodontitis by reducing the levels of pro-inflammatory mediators, influencing the oral microbiota, and alleviating histological changes in periodontal tissue.

## Data Availability

The datasets used and/or analyzed during the current study are available from the corresponding author on reasonable request.
